# Reduced crying in term infants fed high beta-palmitate formula: a double-blind randomized clinical trial

**DOI:** 10.1186/1471-2431-14-152

**Published:** 2014-06-19

**Authors:** Ita Litmanovitz, Fabiana Bar-Yoseph, Yael Lifshitz, Keren Davidson, Alon Eliakim, Rivka H Regev, Dan Nemet

**Affiliations:** 1 Neonatal Department Meir Medical center, (Tchernichovsky 59), Kfar Saba (4428164), Israel; 2 Sackler School of Medicine, Tel-Aviv University, Tel Aviv (6997801), Israel; 3 Infant Nutrition R&D, Enzymotec LTD, Sagi 2000 Industrial Park, Migdal HaEmeq (23106), Israel; 4 Robert H. Smith Faculty of Agriculture, Food and Environment, The Hebrew University of Jerusalem, Rehovot (76100), Israel; 5 Pediatric Department Meir Medical center, (Tchernichovsky 59), Kfar Saba (4428164), Israel

**Keywords:** Beta-palmitate, sn-2 palmitate, Infant formula, Crying, Stool characteristics, Breastfeeding

## Abstract

**Background:**

Beta-palmitate (sn-2 palmitate) mimics human milk fat, enabling easier digestion.

Therefore, we hypothesized that infants consuming high beta-palmitate formula would have more frequent, softer stools and reduced crying compared to infants consuming low beta-palmitate formula.

**Methods:**

Formula-fed infants were randomly assigned to receive either (1) formula with high beta-palmitate (HBP, n = 21) or (2) regular formula with a standard vegetable oil mix (LBP, n = 21). A matched group of breastfed infants served as a reference (BF, n = 21). Crying and stool characteristics data were recorded by the parents for 3 days before the 6- and 12-week visits.

**Results:**

We found no significant differences in the stool frequency or consistency between the two formula groups. The percentage of crying infants in the LBP group was significantly higher than that in the HBP and BF groups during the evening at 6 weeks (88.2% vs. 56.3% and 55.6%, p < 0.05) and during the afternoon at 12 weeks (91.7% vs. 50.0% and 40%, p < 0.05). The infants fed HBP had significantly shorter crying durations when compared with infants fed LBP formula (14.90 ± 3.85 vs.63.96 ± 21.76 min/day, respectively; p = 0.047).

**Conclusions:**

Our study indicates that consumption of a high beta-palmitate formula affects infant crying patterns during the first weeks of life. Comparable to breastfeeding, it reduced crying duration and frequency, primarily during the afternoon and evening hours, thereby improving the well-being of formula-fed infants and their parents.

**Trial registration:**

NCT00874068.

Registration date March 31, 2009

## Background

Crying is a basic, instinctive response governed by basic neuro-chemical mechanisms that are similar to those that control feeding [1]. Although crying is a common spontaneous behavior, it can induce parental concern, which often results in requests for assistance from health services [1]. Most infants follow a universal crying pattern during the first few months of life, in which crying peaks at 6 weeks of age and then declines until 3 months of age [1-6]. Crying also has a typical diurnal pattern: 40% of crying episodes occur between 1600 and 2200 h, and after the third month of life, crying episodes are more distributed throughout the day. Infant crying is usually believed to be related to abdominal discomfort, whereas crying for more than 3 hours per day (cumulative), without relation to a medical problem, is termed infantile colic [7]. The prevalence of abdominal discomfort, colic, and their associated potential effects demonstrates the importance of identifying determinants of infant crying behavior, particularly for determinants that can be modified (e.g., feeding choice) [2].

Human milk is the optimal choice for the vast majority of infants. In human breast milk and most infant formulas, approximately 50% of the dietary calories come from fat, with more than 98% in the form of triglycerides (TGs) [8,9]. Infants easily absorb human milk fat despite its high content of saturated fatty acids (45% of total fatty acids), primarily palmitic acid (C16:0), of which 70-75% is esterified at the sn-2 (beta) position of the TG [8]. This configuration is characterized by a unique, highly specific, positional distribution on the glycerol backbone observed only in TG synthesis in the mammary gland [10]. Studies have shown that palmitic acid is absorbed from human milk as an sn-2 monoacylglycerol [11] and is conserved in that form through digestion, absorption, and chylomicron triacylglycerol synthesis [12]. In contrast, palmitic acid, which is present in the vegetable oils that are commonly used in the manufacturing of infant formulas, is mainly (>80%) esterified to the sn-1 and sn-3 positions [13] and is therefore poorly absorbed by an infant’s intestine. The main cause of this low absorption is the high tendency of palmitic acid esterified at the sn-1 and sn-3 positions to create complexes with dietary minerals, such as calcium [14]. These complexes, known as fatty acid soaps, are insoluble, indigestible, and positively related to stool hardness [15]. The formation of calcium soaps may partly explain the substantial differences in bowel habits and stool consistency between breast- and formula-fed infants [15-17].

The goal of this study was to compare the effects of high-sn-2 palmitate (beta-palmitate) and low-sn-2 palmitate formulas on infant crying patterns and stool characteristics. We hypothesized that infants who consumed high beta-palmitate formula would demonstrate reduced crying and more frequent, softer stools compared to infants consuming low beta-palmitate formula.

## Methods

### Subjects and study design

The study was a single-center, randomized, double-blind, placebo-controlled study [18]. Healthy term infants whose weights were appropriate for gestational age (AGA) were eligible for the study. Infants were excluded from the study if they had been diagnosed with congenital, chromosomal, or metabolic disorders. Subjects were enrolled within their first 2 weeks of life to one of two formula groups if their mother made an unequivocal decision not to breastfeed or to a matched reference group of term breastfed infants. Parents provided written informed consent prior to enrollment. Formula-fed infants were randomly assigned (using a computerized automatic randomization system) to receive either (1) formula with a high level of beta-palmitate (HBP group) consisting of 44% of palmitic acids esterified at the midpoint position of the glycerol backbone (INFAT®, Advanced Lipids) or (2) a regular infant formula (LBP Control group) with a standard vegetable oil mix consisting of 14% of palmitic acids esterified to the midpoint position of the glycerol backbone. Both of the preparations were commercial formulas produced by Materna Laboratories (Maabarot, Israel) under the same conditions using minerals and vitamins from the same batches, resulting in formulas that were identical except for their fatty acid structural distribution. Formulas were packed in identical and unmarked boxes, and the personnel caring for the infants and mothers were blinded to their contents. The formulas did not contain pre- or probiotic components.

This study was conducted according to the principles of the Declaration of Helsinki and good clinical practice. The protocol was approved by the Ethics Committee of Meir Medical Center, Kfar-Saba, Israel, and by the Israeli Ministry of Health.

### Anthropometric measurements

Anthropometric measurements, performed at birth and at 6 and 12 weeks, by a trained technician (who was blinded to the study group assignment) included the following:

Body Weight: the mean of three measurements on an electronic infant scale (Model 20 Tabletop Infant Scale, Olympic Medical, Seattle, WA).

Body Length: the mean of two measurements of recumbent crown-heel length on a length board (O’Leary Premier Length Board, Ellard Instrumentation Ltd., Monroe, WA) to the nearest 0.1 cm.

Head Circumference: fronto-occipital head circumference measured with a standard 1-cm wide measuring tape to the nearest 0.1 cm.

### Parent questionnaires

Data on crying and stool characteristics were reported by the parents, who used 24-hr behavior diaries for 3 days before the infants’ 6- and 12-week visits. The reports also included (1) the volume of formula used in feedings and (2) stool characteristics, including color (yellow, green, brown, and black), frequency and consistency (hard, mushy, or watery). Crying episodes of more than 5 minutes were reported. The parents reported the duration, time of the day, and their assumption of what caused the crying. The mean number of crying periods per day, the mean crying duration per day, and the pattern of crying during the day were calculated. The pattern of crying during the day was analyzed at 6 and 12 weeks by calculating the percentage of crying infants and the total length of crying during each 6-hour period of the day, where morning = 6 am–noon, afternoon = noon–6 pm, evening = 6 pm–midnight, and night = midnight–6 am.

### Statistical analysis

The baseline characteristics of the mothers and infants in the HBP and LBP formula groups were compared with pairwise *t*-tests for scale outcomes and pairwise chi-square tests for nominal outcomes. The crying parameters were tested at 6 and 12 weeks for all infants using the available accurate crying data at each time point. All parameters of the formula groups were compared to those of the BF group (for reference). Statistical analyses were conducted with SPSS v20 (SPSS, Inc., Chicago, IL). The data are expressed as the means ± SEMs. Statistical significance was inferred at p < 0.05.

## Results

### Study population

**Figure 1:**
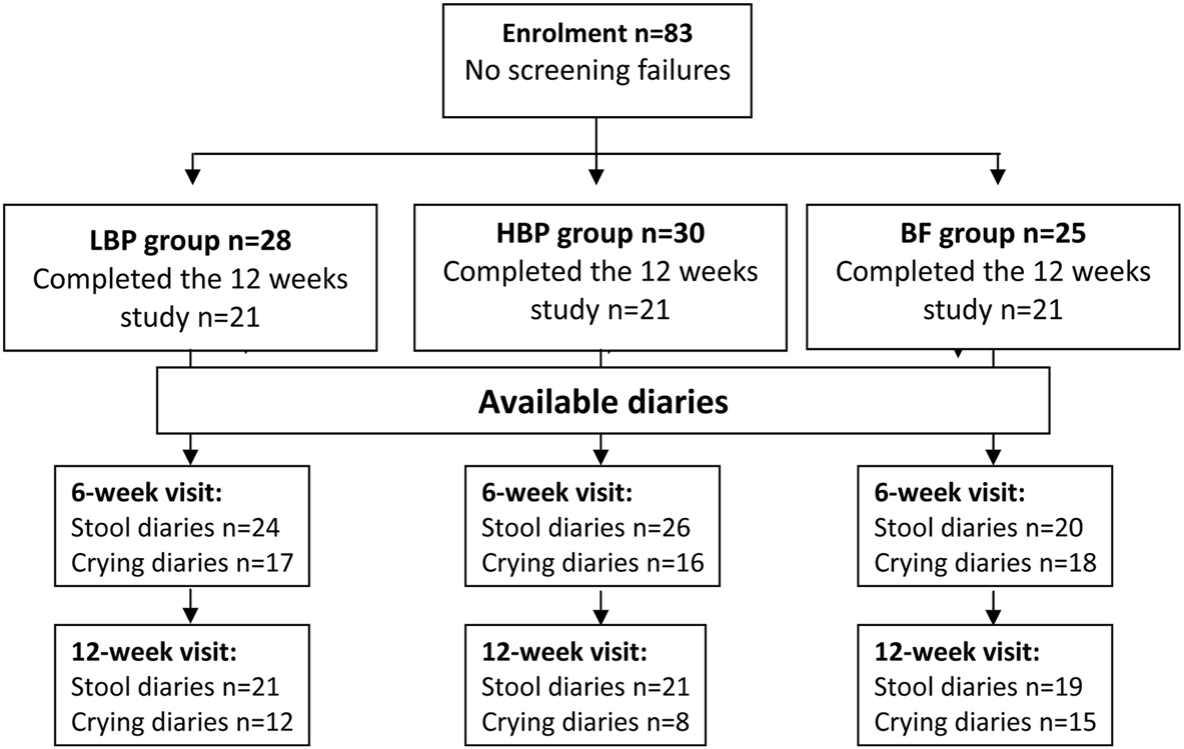
Participant flow diagram.

Between March 2009 and Nov 2011, eighty-three infants born at the Meir Medical Center were enrolled in the study, including 58 formula-fed infants and 25 exclusively breastfed infants. The formula-fed infants were randomly assigned to receive either HBP formula (n = 30) or LBP formula (n = 28). The attrition rate at the end of the study period was 21%, and it was equally distributed between the three groups (Figure 1); the sample of non-completers was matched to those who completed the study.

The maternal and infant baseline characteristics are presented in Table 1. No significant differences were observed between the two formula groups; however, the breastfeeding mothers had significantly higher education levels (>12 years). The infants in the breastfed group were born at a significantly greater gestational age. Moreover, the infants in the breastfed group were all singletons, whereas twins represented 21.4% and 26.7% of the infants in the LBP and HBP formula groups, respectively. The mean formula consumption was comparable between the two formula groups both at 6 weeks (177 vs. 178 ml/kg/day, in the HBP and LBP groups, respectively) and at 12 weeks (143 vs. 139 ml/kg/day).

**Table 1:** Baseline maternal and infant demographic characteristics

	**LBP**	**HBP**	**BF**
	**(n = 28)**	**(n = 30)**	**(n = 25)**
Maternal age, years	33.3 ± 0.8	32.3 ± 0.6	31.6 ± 0.8
Maternal education (% of women with >12 years of education)	53.6%*	60.0%*	92.0%
Primigravida (%)	21.4%	16.7%	32.0%
Gestational age, weeks	39.1 ± 0.2*	39.1 ± 0.2*	39.7 ± 0.2
Type of delivery (% vaginal)	57.1%	72.4%	68.0%
Twins (%)	21.4%*	26.7%*	0.0%
Gender (% male)	46.4%	60.0%	68.0%
Birth weight, kg	3.2 ± 0.5	3.1 ± 0.6	3.4 ± 0.4

Mean ± SEM.

*p < 0.05 compared to BF.

Anthropometric data at baseline and at 6 and 12 weeks postnatal are presented in Table 2. All infants maintained normal growth throughout the study period, and by the end of the study (i.e., 12 weeks postnatal), no significant differences were observed between the groups. No significant differences were found between the two formula-fed groups in mean weight, length, and head circumference during the study visits (baseline and 6 and 12 weeks postnatal).

**Table 2:** Anthropometric parameters measured at baseline and at 6 and 12 weeks postnatal

	**LBP**	**HBP**	**BF**
**Baseline**			
Weight (Kg)	3.1 ± 0.1	3.0 ± 0.1	3.2 ± 0.1
Length (cm)	49.1 ± 0.4	49.2 ± 0.5	50.3 ± 0.5
Head circumference (cm)	34.1 ± 0.3	34.0 ± 0.3*	34.9 ± 0.3
**6 weeks**			
Weight (Kg)	4.6 ± 0.1*	4.6 ± 0.1*	5.0 ± 0.1
Length (cm)	55.0 ± 0.5*	55.1 ± 0.5*	56.8 ± 0.5
Head circumference (cm)	37.3 ± 0.4*	37.3 ± 0.3*	38.3 ± 0.2
**12 weeks**			
Weight (Kg)	5.9 ± 0.2	5.9 ± 0.2	6.2 ± 0.2
Length (cm)	59.8 ± 0.5	59.8 ± 0.6	60.1 ± 0.4
Head circumference (cm)	40.1 ± 0.3	39.5 ± 0.4	39.9 ± 0.4

Mean ± SEM.

*p < 0.05 compared to BF.

### Stool characteristics

Stool characteristics were evaluated at 6 and 12 weeks postnatal based on the 3-day diaries completed by parents prior to each visit (Table 3). Stool characteristics were evaluated according to the mean number of stools per day, stool consistency (1 = hard, 2 = soft, 3 = watery), and the percentage of infants who had hard stools.

**Table 3:** Stool characteristics at 6 and 12 weeks postnatal

	**LBP**	**HBP**	**BF**
**6 weeks**			
Stools per day	2.0 ± 0.2*	2.4 ± 0.3*	3.8 ± 0.5
Stool consistency score	1.9 ± 0.1*	1.9 ± 0.0*	2.4 ± 0.1
Percent of infants with hard stools	16.7%	23.1%	5.0%
**12 weeks**			
Stools per day	1.6 ± 0.2*	1.4 ± 0.1*	3.2 ± 0.5
Stool consistency score	2.0 ± 0.1*	2.0 ± 0.1*	2.4 ± 0.1
Percent of infants with hard stools	23.8%*	14.3%	0.0%

Mean ± SEM or percent.

*p < 0.05 compared to BF.

The BF infants had significantly higher stool frequencies (mean no. of stools per day) than the infants in both the HBP and LBP formula groups at both 6 weeks postnatal (3.8, 2.0, and 2.4, respectively, p < 0.05) and 12 weeks postnatal (3.2, 1.6, and 1.4, respectively, p < 0.01). The breastfed infants also had softer stools, with significantly higher mean stool consistency scores at both 6 weeks postnatal (2.4, 1.9, and 1.9, respectively, p < 0.05) and 12 weeks postnatal (2.4, 2.0, and 2.0, respectively, p < 0.05). No significant differences in stool frequencies or consistencies were observed between the two formula groups. At 6 weeks, hard stools were reported for only 5% of the BF infants, compared with 16.7% and 23.1% of the infants in the LBP and HBP groups, respectively. At 12 weeks, however, a significant reduction in hard stools was observed for the HBP group but not the LBP group, and the difference remained significant only between the BF group and the LBP group (0.0% vs. 23.8%, p = 0.023; Table 3).

### Crying duration

**Figure 2:**
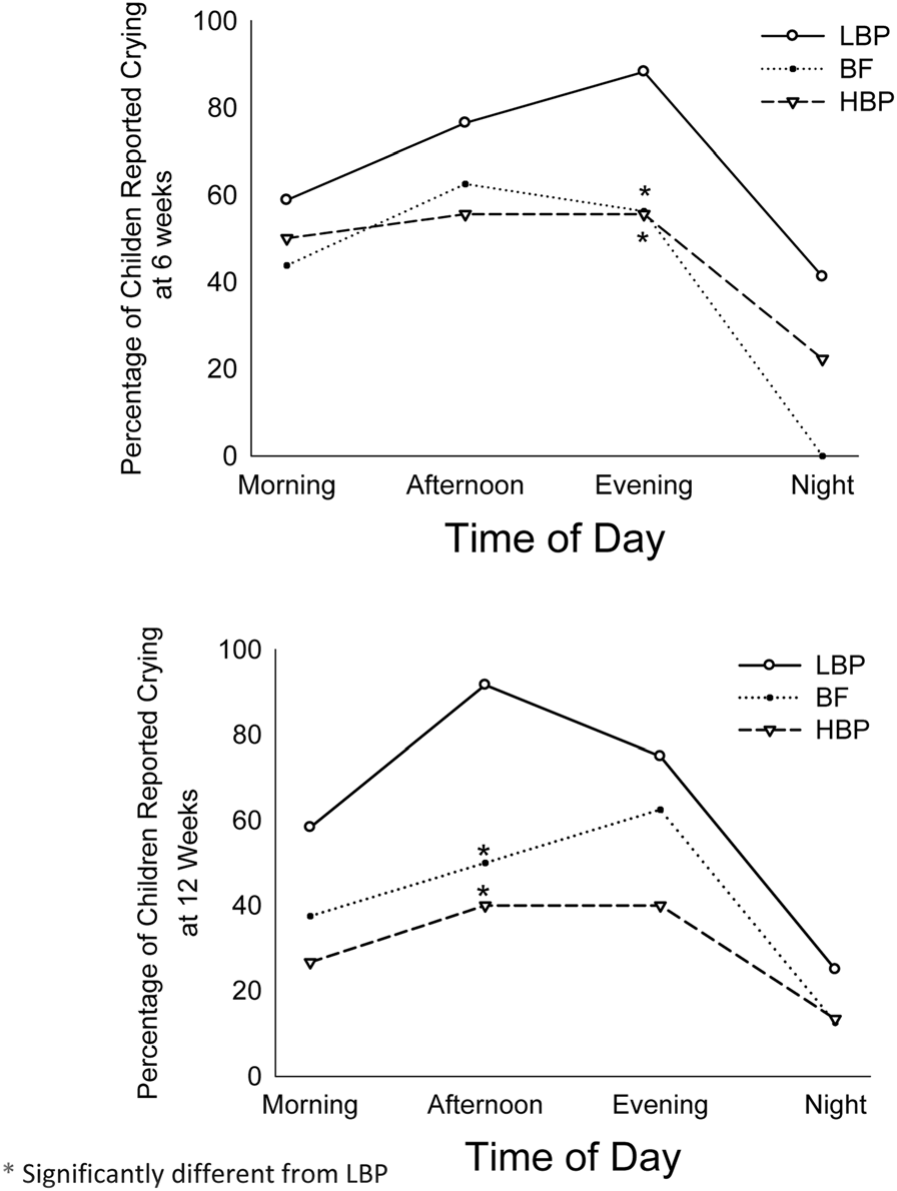
Diurnal pattern of crying in LBP formula-fed, HBP formula-fed, and breastfed infants at 6 (Upper panel) and 12 (Lower panel) weeks postnatal.

**Figure 3:**
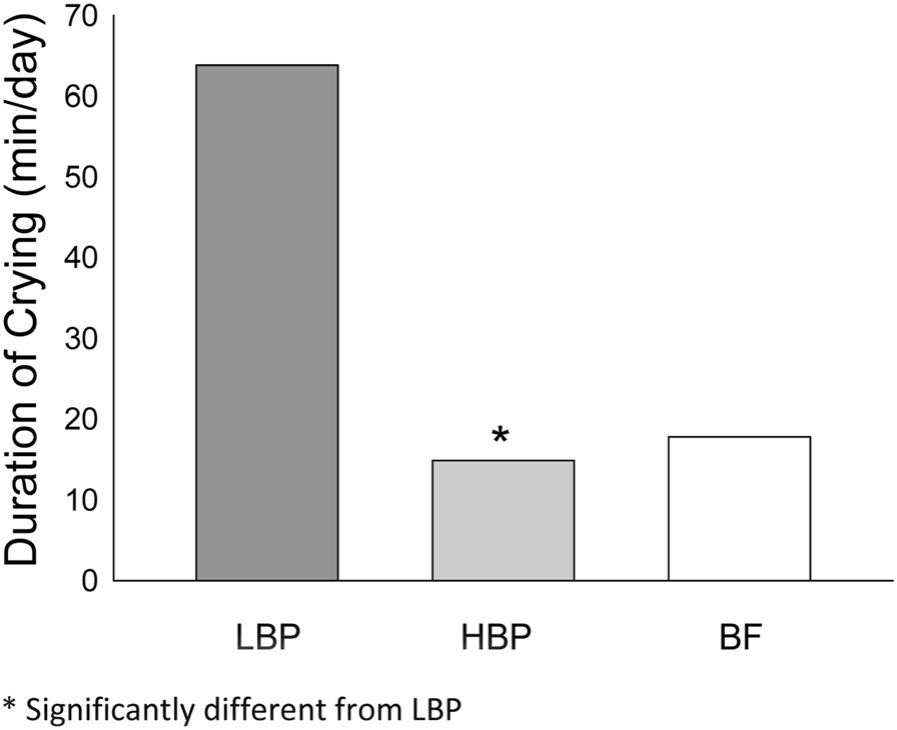
**Total daily crying in LBP formula-fed, HBP formula-fed, and breastfed infants at 12 weeks of age.** Data are means ± standard error.

Figure 2 shows the percentages of infants with reported crying events at each period of the day. The percent of crying infants peaked in the afternoon-evening and declined at night. The crying pattern in the HBP group was comparable to that of the BF group at both 6 and 12 weeks. The percentage of crying infants in the LBP group was higher than that of both the HBP and the BF groups during the evening at 6 weeks (88.2% vs. 56.3% and 55.5%, respectively; p < 0.05) and during the afternoon at 12 weeks (91.7% vs. 50.0% and 40%, respectively; p < 0.05).At 12 weeks, the total duration of crying in HBP infants was comparable to that of BF infants. Infants consuming LBP formula had a significantly longer mean total crying duration when compared to the HBP infants (63.96 ± 21.76 vs. 14.90 ± 3.85 min/day; p = 0.047; Figure 3). Infants in the HBP group cried less frequently than infants in the LBP group at 12 weeks postnatal (0.3 vs. 0.8 periods/day, p = 0.028).

## Discussion

This randomized controlled study showed that term infants who consumed formula with a high beta-palmitate content (HBP) cried less than infants who consumed LBP formula. Additionally, the study showed that the consumption of formula with beta- palmitate improved infant crying patterns during the day. The crying duration and patterns in the HBP group were comparable to those of breastfed infants, and the percentage of crying infants was significantly lower in the HBP group than in the LBP group during the evening and night hours.

Previous studies have shown that the daily duration of crying and fussing in infants increases until 6 weeks of age and then decreases progressively [1-6,19]. Our study showed similar peaks of daily crying at 6 weeks for all groups. Harrison et al. found a 50% reduction in daily crying duration from 6 to 12 weeks [20]; however, in their study, the type of feeding was not specified. Our study revealed a similar reduction in daily crying duration, both in the BF group (50%) and the HBP group (80%). In contrast, a small increase in crying time was observed in the LBP group.

Crying commonly clusters in the afternoon and evening [21,22]. We also observed this peak in this study for all three groups; however, we found that infants in the HBP group cried less during the afternoon and evening hours and had significantly greater reductions in crying duration in the afternoon between 6 and 12 weeks postnatal.

The effects of breastfeeding versus formula feeding on infant crying are controversial. Many studies support breastfeeding as an effective method of calming infants due to its emotional impacts [23]. Kennedy et al. [16] monitored crying duration in infants who consumed formula containing high versus low levels of beta-palmitate. In contrast to our study, they did not find differences in crying between the groups. Savino et al. [24] evaluated the efficacy of formula that was partially hydrolyzed with prebiotic oligosaccharides (OSs) and contained a high level of beta palmitate. The authors found significant differences in crying that were primarily due to a significant reduction in the afternoon crying duration between 6 and 12 weeks postnatal. In their study, however, the formulas contained several different components (partially hydrolyzed whey proteins, prebiotic OSs, and beta palmitate); therefore, the relative contribution of the HBP component could not be determined.

Crying behavior in infants is a complex phenomenon that includes spontaneous "endogenous" crying and “distress-related” crying, which can be linked to separation, hunger, or other physical distresses; therefore, several mechanisms can explain the positive effect of HBP on crying. One mechanism can be attributed to the effect that HBP has on stool characteristics. In this study, HBP-fed infants demonstrated more favorable stool characteristics than LBP-fed infants at 12 weeks. Improvement of stool characteristics is one of the well-known benefits of beta-palmitate [16,25,26]. In a previous study, we showed that high beta-palmitate formula beneficially affected infant gut microbiota by increasing the lactobacillus and bifidobacteria counts in stools [27]. This is another possible mechanism because changes in gut microflora induced by probiotic supplementation likely improve infant colic [21].

Animal studies suggest that diet can alter endogenous crying by modifying neuro-endocrine metabolism in the gut (e.g., through the endocannabinoid system, which is associated with a variety of functions, including pain and circadian rhythm). Using a rat model, Banni et al. [27] showed that a diet rich in beta-palmitate led to higher efficiency and enhanced endocannabinoid biosynthesis.

One possible limitation of our study is that we relied on the parents to record crying measurements. Parent diaries of infant crying have become the standard and most widely used tool for studying infant crying [1,28]; however, this method is still subjective and requires a high degree of parental cooperation. In this study, this limitation was partially overcome by the double-blind nature of our study. We were also limited in this study because crying is known to be highly affected by an infant’s temperament [2], which could have biased our results. We could not, however, overcome this limitation because we did not assess the temperaments of the infants included in this study.

## Conclusions

Our study indicates that high beta-palmitate (sn-2 palmitate) formula affects infant crying patterns during the first weeks of life. Comparable to breastfeeding, high beta-palmitate reduced crying duration and frequency, primarily during the afternoon and evening hours, thereby improving the well-being of formula-fed infants and their parents.

## Abbreviations

HPB: High beta-palmitate; LBP: Low (regular) beta-palmitate; BF: Breastfed; TG: Triglycerides; AGA: Appropriate for gestational age; Oss: Oligosaccharides.

## Competing interests

Fabiana Bar-Yoseph and Yael Lifshitz are Enzymotec employees and therefore declare a relevant financial interest in this work.

## Authors' contributions

IL participated in the study design, follow-up, and data collection, analysis and interpretation; she has also been involved in the drafting and revision of the manuscript (MS). FB and YL contributed to the design of the study and to the revision of the MS. KD was responsible for the collection of the data and contributed to its analysis and interpretation. AE contributed to the design, analysis and interpretation of the data and to the revision of the MS. RR was involved in data analysis and interpretation, as well as in the revision of the MS. DN contributed to the study conception and design and the analysis of the data. He was involved in drafting the MS and preparing its final version. All the authors approved the final version of the MS.

## Pre-publication history

The pre-publication history for this paper can be accessed here:

http://www.biomedcentral.com/1471-2431/14/152/prepub
